# Four Immune-Related Long Non-coding RNAs for Prognosis Prediction in Patients With Hepatocellular Carcinoma

**DOI:** 10.3389/fmolb.2020.566491

**Published:** 2020-12-08

**Authors:** Muqi Li, Minni Liang, Tian Lan, Xiwen Wu, Wenxuan Xie, Tielong Wang, Zhitao Chen, Shunli Shen, Baogang Peng

**Affiliations:** ^1^Department of Liver Surgery, The First Affiliated Hospital of Sun Yat-sen University, Guangzhou, China; ^2^Center of Surgery and Anaesthiology, The First Affiliated Hospital of Sun Yat-sen University, Guangzhou, China; ^3^Department of Pancreatobiliary Surgery, The First Affiliated Hospital of Sun Yat-sen University, Guangzhou, China; ^4^Organ Transplant Center, First Affiliated Hospital of Sun Yat-sen University, Guangzhou, China; ^5^Guangdong Provincial Key Laboratory of Organ Donation and Transplant Immunology, Guangzhou, China; ^6^Guangdong Provincial International Cooperation Base of Science and Technology (Organ Transplantation), Guangzhou, China

**Keywords:** hepatocellar carcinoma, long noncoding (lnc) RNAs, prognosis, survival, bioinformation analysis

## Abstract

**Background:**

Long non-coding RNA (LncRNA) plays an important role in the occurrence and development of hepatocellular carcinoma (HCC). This study aims to establish an immune-related LncRNA model for risk assessment and prognosis prediction in HCC patients.

**Methods:**

Hepatocellular carcinoma patient samples with complete clinical data and corresponding whole transcriptome expression were obtained from the Cancer Genome Atlas (TCGA). Immune-related genes were acquired from the Gene Set Enrichment Analysis (GSEA) website and matched with LncRNA in the TCGA to get immune-related LncRNA. Least Absolute Shrinkage and Selection Operator (LASSO) regression was used for screening the candidate LncRNAs and calculating the risk coefficient to establish the prognosis model. Patients were divided into a high-risk group and a low-risk group depending on the median risk score. The reliability of the prediction was evaluated in the validation cohort and the whole cohort. GSEA and principal component analysis were used for function evaluation.

**Results:**

A total of 319 samples met the screening criteria and were randomly distributed across the training cohort and the validation cohort. After comparison with the IMMUNE_RESPONSE gene set and the IMMUNE_SYSTEM_PROCESS gene set, a total of 3094 immune-related LncRNAs were screened. Ultimately, four immune-related LncRNAs were used to construct a formula using LASSO regression. According to the formula, the low-risk group showed a higher survival rate than the high-risk group in the validation cohort and the whole cohort. The receiver operating characteristic curves data demonstrated that the risk score was more specific than other traditional clinical characteristics in predicting the 5-year survival rate for HCC.

**Conclusion:**

The four-immune-related-LncRNA model can be used for survival prediction in HCC and guide clinical therapy.

## Introduction

Hepatocellular carcinoma (HCC) is one of the most lethal cancers in the world. Although medical technology has improved in recent years, the 5-year survival rate and mortality rate of HCC have not significantly improved (Siegel et al., 2019). Surgery is the curative treatment for HCC, but less than 30% of patients have the opportunity of undergoing a radical operation upon diagnosis (European Association for the Study of the Liver et al., 2018). Therefore, the exploration of non-operative methods and an evaluation system for prognosis are urgently needed.

In addition to surgical procedures such as hepatectomy, radiofrequency ablation, and liver transplantation, non-operative therapies including targeted therapy with tyrosine kinase inhibitors and immunotherapy with programmed death 1 (PD-1) or programmed death 1 ligand (PD-L1) antibodies are now the mainstay treatments for advanced HCC. It has been proved that an immune response disorder in the cancer microenvironment plays an important role in the tumor development (Teng et al., 2015). Because of the general tolerance and immunosuppressive environment of the liver, once HCC has developed, this mechanism might protect the tumor and assist in its growth, further epithelial-mesenchymal transformation, and the development of a true tumor microenvironment, enhancing suppressive immunity such as the expression of PD-L1 and protecting cancer cells (Kubes and Jenne, 2018). Many cancers suppress T cell attacks by overexpressing inhibitory ligands to avoid suppression of the tumor by the immune system. Therefore, the low number of T cells in patients with HCC and their vulnerability to damage may be the reasons leading to the development of HCC (Jia et al., 2015). Over the past few years, great progress was made in the application of immune checkpoint inhibitors (ICI) such as cytotoxic T lymphocyte antigen 4 (CTLA-4), PD-1, and PD-L1 for the clinical treatment of HCC (Sprinzl and Galle, 2017). The CTLA-4 blocker tremelimumab was effective in approximately 17.6% of HCC patients with a hepatitis C virus infection, while the progression time was about 6.48 months (Sangro et al., 2013). In combination with local tumor ablation, tumor-associated antigens (TAAs) can be released from apoptotic or necrotic liver cancer tissue, which accelerates the activation of tumor-oriented T cells and immune synergism (Mizukoshi et al., 2011). On the other hand, the assertiveness of the PD-1 pathway can change the duration of T cell-APC or T cell-target cell contact and decrease the number of T-reg cells in the tumor to enhance the anti-tumor immune response (Fife et al., 2009; Francisco et al., 2009). Although PD1 antibody-based immunotherapy has made considerable strides in the treatment of HCC, there are still some deficiencies in the clinical application of these drugs. First, these drugs may cause complications such as diarrhea, rashes, lung disease, and even lethal complications (Zhong et al., 2016; Zhang X. et al., 2019). Second, their high price is beyond the reach of many families. Third, PD-1 antibody monotherapy appears to be effective only in a very small subset of patients, with no effect in the majority of patients (Shivaji et al., 2019). Hepatocellular carcinoma has multiple immunophenotypes, which underscores the importance of further research on immune cell interaction in HCC and the development of individualized immunotherapy drugs (Hilmi et al., 2019; Llovet et al., 2019). The expression of PD-1 may be involved in the screening of sensitive populations with higher levels of PD-1/PD-L1 who have better histologic response rates and progression-free survival (PFS) and overall survival (OS) after immunotherapy (Patel and Kurzrock, 2015). Accurate screening criteria to maximize the benefits of immunotherapy are of great significance.

Long non-coding RNAs (LncRNAs) are a class of non-coding RNAs of more than 200 nucleotides in length (Ma et al., 2017). Long non-coding RNA plays a crucial part in gene expression, cell growth, differentiation, and other biological processes (Klec et al., 2019). At the same time, genome sequencing revealed that the expression of LncRNAs is different in different kinds of cancers (Jing et al., 2018; Nanni et al., 2020; Yn et al., 2020). In addition, studies indicated that LncRNAs may play a role in inhibiting or promoting the occurrence and development of cancers (He et al., 2014). Meanwhile, LncRNA may be a marker of tumor prognosis or an immunotherapy target (Li et al., 2020). Studies show that LncRNA plays a broad regulatory role in functional activities such as immune response, tumorigenesis, and tumor progression (Bhan et al., 2017; Yu et al., 2018). It was found that some of the immune-related LncRNAs regulate the biological behavior in diseases by regulating the immunosuppressive activity of bone marrow mesenchymal stem cells, the expression level of T/B cells and natural killer cells, etc. (Wang et al., 2015; Zheng et al., 2019; Ahmad et al., 2020). Long non-coding RNAs have been studied using high-throughput sequencing and bioinformatics methods, and their prognostic value has been evaluated by creating formulas for models (Wang et al., 2018; Yan et al., 2019; Shen et al., 2020). However, the relationship between immune-related LncRNAs and HCC prognosis has been studied less extensively.

In this study, we try to establish a prognosis model based on immune-related LncRNAs for predicting the prognosis and guiding the application of immunotherapy in HCC.

## Materials and Methods

### Patient Cohort and Grouping

RNA sequencing data and corresponding clinical information of HCC patients were obtained from the Cancer Genome Atlas (TCGA)-Liver hepatocellular carcinoma (LIHC) of the TCGA database^1^ (Gao et al., 2013; Li et al., 2015). All patients were diagnosed with HCC. Patients were excluded if their follow-up time was less than 30 days or their pathological stage was unclear. Overall, 319 patients met the screening criteria. The patients were randomly divided into a training cohort (*n* = 160) and a validation cohort (*n* = 159). A multi-LncRNA model of prognosis was established using the training cohort, and the validation cohort was used to test the predictive power of the equation. There is no difference in clinical characteristics between the training cohort and validation cohort.

### The Expression of Genes and Relative Immune LncRNAs in the TCGA-LIHC Cohort

We integrated the human gene annotation file from the Gene Expression Omnibus (GEO) database and Ensemble website. Based on ensemble-ID, the genes obtained from the TCGA database were identified as protein-coding genes or non-coding genes. We then compared the contents with the data retrieved from the TCGA, enabling us to distinguish between LncRNA and other kinds of RNA from all gene expression (Zhang et al., 2012). The original data were processed by FPKM (fragments per kilobase million). This normalization method can avoid errors caused by differences in gene length and sequencing depth. The data acquired from the dataset were consolidated and processed by the “limma” package in R. In addition, 7009 LncRNAs were selected from the TCGA-LIHC project. A total of 331 immune-related genes were obtained by matching the gene sets from the TCGA with the IMMUNE_RESPONSE gene set (M19817) and the IMMUNE_SYSTEM_PROCESS gene set (M13664) in the molecular characterization database V7.0^2^ (Subramanian et al., 2005; Wang et al., 2018; Sun et al., 2020). In total, 3094 immune-related LncRNAs were obtained through correlation analysis between the LncRNA expression and immune gene expression. According to the hazard ratio, the expression of LncRNA could be defined as positive or negative (cor > 0.4 and *P* value < 0.01). For patients with biological replicates, the final expression level was the mean expression of all repeated samples of LncRNA. The LncRNA was excluded from the expression matrix if it was missing in more than 20% of HCC patients, to avoid calculation bias (Yan et al., 2019).

### Construction and Evaluation of an Immune-Related LncRNA Prediction Model

Univariate Cox regression analysis was used to determine the relationship between the level of immune-related LncRNA expression and OS. LncRNAs with a *P* value less than 0.01 are considered as significant prognostic LncRNAs. Least Absolute Shrinkage and Selection Operator (LASSO) Cox regression analysis and the “glmnet” package in R were used to analyze the best candidates and multiple immune LncRNA features for predicting the OS of patients (Zhang M. et al., 2019; Gupta et al., 2020). Based on this model, the LASSO regression coefficients weighted LncRNA expression levels were recruited to calculate the risk score for each patient.

The relationship between clinical features, risk score, and patient OS univariate was calculated by univariate independent prognostic analysis and multivariate independent prognostic analysis. The area under the curve (AUC) and the time-dependent receiver operating characteristic (ROC) curves were assessed using the “survival” package and “survivalROC” package of R language to evaluate the predictive power of the signal for survival prediction. The “heatmap” package was used for acquiring related graphics such as heatmaps, the distribution of risk scores with survival, and statuses of patients dependent on selected immune LncRNA expression. The patients in the training cohort were divided into a high-risk group and a low-risk group based on the mean risk score. The survival differences between the high-risk group and the low-risk group were evaluated by “Kaplan–Meier” and the “survival” package log-rank test (Wei et al., 2019). The method was also applied to the verification in the validation cohort and the whole cohort.

Principal component analysis (PCA) was used to visualize and compare the predictive power of selected immune LncRNAs and all immune LncRNAs and immune genes. Gene Set Enrichment Analysis (GSEA) was employed to predefine the enrichment of immune gene sets and find different functional phenotypes. All analyses were performed by using the R programming language.^3^

### Statistical Analysis

The chi-square test and Student’s *t*-test were adopted to confirm that there is no significant difference in clinical characteristics between the training cohort and validation cohort. Kaplan–Meier survival analysis was performed to compare the survival rate of the high-risk group and low-risk group. The independent prognostic factors in HCC were determined using univariate Cox regression and multivariate Cox regression. SPSS26.0 was used for all statistical analyses. A *P* value <0.05 is considered significant.

## Results

### Expression of LncRNAs in HCC Patients

Based on the screening criteria, 319 patients in TCGA-LIHC were selected with matching complete gene expression. We randomly divided them into a training cohort (*n* = 160) and a validation cohort (*n* = 159). The chi-square test showed no significant difference between the two groups (Table 1).

**TABLE 1:** Clinical factors of patients with ICC.

	Training cohort (*n* = 160)	Validation cohort (*n* = 159)	Whole cohort (*n* = 319)	*P* value
**Clinical factors**				
**Gender**				
Male	49	51	100	
Female	111	108	219	0.810
**Age (years)**				
≤60	80	80	160	
>60	80	79	159	1.000
**Grade**				
G1 + G2	98	100	198	
G3 + G4	62	59	121	0.818
**TNM stage**				
I + II	120	116	236	
III + IV	40	43	83	0.703
**Survival status**				
Alive	114	101	215	
Dead	46	58	104	0.153

We screened out the non-coding RNAs by determining whether the genes in the mRNA matrix encode proteins or not and further sorted out the LncRNA matrix. The correlation test was used between LncRNAs and immune genes, the screening coefficient was set to Cor-Filter >0.4 or <−0.4, while the *P* value was set to less than 0.001 (Zhang Y. et al., 2019). The non-fluctuation LncRNA expression was deleted in the process. We ended up with 3094 immune-related LncRNAs in the training cohort. Most of the genes were significantly upregulated (*n* = 3038) and a small part of the immune-related LncRNAs were downregulated.

### Construction of a Prognostic Model for Survival Prediction in the Training Cohort

Twenty-four LncRNAs were found to be significantly associated with OS in the training cohort by univariate Cox regression. Least Absolute Shrinkage and Selection Operator Cox regression analysis was performed to identify immune-related LncRNA related to prognosis. The eight most relevant prognostic parameters were calculated and cross-validated to facilitate parameter selection. Some parameters were removed if their coefficient was 0 to prevent overfitting (Liu et al., 2020). After excluding LncRNAs with a coefficient less than 0.1, four immune LncRNAs with the closest relationship to prognosis were selected and a risk score formula was developed through linear combination of the expression values of the four LncRNAs adjusted by the LASSO regression coefficient. The risk score is equal to (expression value of AL603839.3^∗^0.15195028612722) + (expression value of MSC-AS1^∗^0.101019611929165) + (expression value of AL031985.3^∗^0.758428456611441) + (expression value of THUMPD3-AS1^∗^0.127206488953884). The risk score for each patient was calculated based on this equation (Table 2).

**TABLE 2:** The four immune LncRNAs screened out by univariate Cox regression and LASSO regression analysis.

LncRNAs	Hazard ratio (95% CI)	*P* value	Coefficient
AL603839.3	2.253 (1.436-3.536)	<0.001	0.151950286127222
MSC-AS1	2.088 (1.414-3.083)	<0.001	0.101019611929165
AL031985.3	7.225 (3.068-17.014)	<0.001	0.758428456611441
THUMPD3-AS1	2.861 (1.662-4.924)	<0.001	0.758428456611441

**FIGURE 1:**
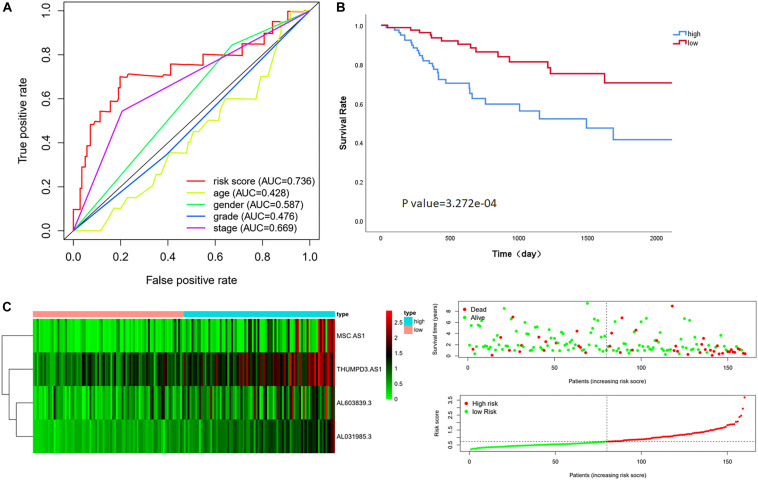
Performance evaluation of the four immune LncRNAs for survival prediction in the training cohort. **(A)** ROC curve for 5 years of OS. **(B)** Kaplan–Meier survival curve for high-risk and low-risk groups divided by the median. **(C)** Heatmaps, distribution of risk scores with survival, and statuses of patients depend on selected immune LncRNA expression.

The median risk score in the training cohort was recorded as the dividing point that is used to divide the cohort into a high-risk group and a low-risk group. Kaplan–Meier analysis showed that the OS in high-risk patients was significantly lower than that in low-risk patients (*P* value = 3.272e-04). The median survival time was 564 days and 1145 days in each group, respectively. The 5-year survival rates in the high-risk and low-risk groups were 41.5 and 70.5%, respectively (Figure 1C). The specific risk score distribution, survival status of the patients, and the selected four prognostic expression profiles also showed that a high expression of the four immune-related LncRNAs correlated with a poor prognosis (Figure 1B).

### Comparison Between Four LncRNAs Signature and Clinical Characteristics

Multivariate Cox regression analysis showed that the risk score was associated with OS in the training cohort after adjusting for age, gender, grade stage, and Tumor, Node, metastasis (TNM) stage [HR = 4.863, 95% CI (3.004–7.872), *P* value < 0.001] (Ochenduszko et al., 2017). Although we found that the TNM stage also can evaluate the prognosis as an independent factor significantly in multivariate regression, the AUC of the ROC analysis predicting 5-year OS showed that the risk score was highest at 0.736, indicating that it is more accurate than other traditional clinical parameters in survival prediction (Figure 1A).

### Testing in the Validation Cohort and the Whole Cohort

**FIGURE 2:**
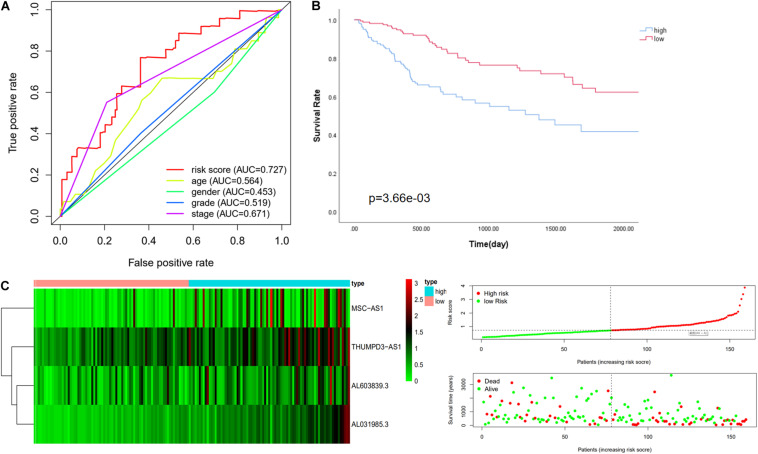
Performance evaluation of the four immune LncRNA for survival prediction in the validation cohort. **(A)** ROC curve for 5 years of OS. **(B)** Kaplan–Meier survival curve for high-risk and low-risk groups divided by the median. **(C)** Heatmaps, distribution of risk scores with survival, and statuses of patients depend on selected immune LncRNA expression.

The risk score was confirmed to be an independent prognostic factor in the training cohort. Further verification was done in the validation cohort and the whole cohort to confirm the usability of the model. The expression value of immune-related LncRNAs of patients in the validation cohort was used to calculate the risk score depending on the equation. The median value of the risk score in the training cohort (risk score = 0.728) was adopted to divide the validation cohort into a low-risk group (*n* = 78) and a high-risk group (*n* = 81). First of all, univariate analysis and multivariate analysis showed that the risk score correlated with OS after adjustment for age, gender, grade, and TNM stage (Table 3; Scheiner et al., 2019). Consistently with this finding, Kaplan-Meier analysis revealed that the OS in high-risk patients was significantly lower than in low-risk patients (*P* value = 3.66e-03, Figure 2B). The 5-year survival rates in the high-risk and low-risk groups were 33.0 and 49.5%, respectively. The specific risk score distribution, survival status of the patients, and the selected four prognostic expression profiles also showed the same result as in the training cohort (Figure 2C). The ROC analysis indicated that the risk score is the best signature (AUC = 0.727) among the clinical characteristics (Figure 2A).

**TABLE 3:** Univariate and multivariate Cox regression analysis in each cohort.

Variables	Univariate analysis		Multivariate analysis	
	HR (95% CI)	*P* value	HR (95% CI)	*P* value
**Training cohort (*n* = 160)**				
Age (>60/≤60)	1.007 (0.984–1030)	0.560	1.000 (0.976–1.025)	0.998
Gender (female/male)	1.202 (0.629–2.296)	0.577	1.491 (0.745–2.982)	0.259
Grade (G1 + G2/G3 + G4)	1.015 (0.558–1.847)	0.961	0.717 (0.374–1.374)	0.316
TNM stage (S1 + S2/S3 + S4)	3.332 (1.851–5.999)	<0.001	3.733 (2.005–6.953)	<0.001
Risk score (low/high)	4.519 (2.844–7.182)	<0.001	4.863 (3.004–7.872)	<0.001
**Validation cohort (*n* = 159)**				
Age (>60/≤60)	1.007 (0.984–1.030)	0.560	0.997 (0.974–1.021)	0.805
Gender (female/male)	1.202 (0.629–2.296)	0.577	1.008 (0.542–20185)	0.813
Grade (G1 + G2/G3 + G4)	1.015 (0.558–1.847)	0.961	0.699 (0.361–1.352)	0.288
TNM stage (S1 + S2/S3 + S4)	3.332 (1.851–5.999)	<0.001	3.365 (1.808–6.263)	<0.001
Risk score (low/high)	3.627 (2.510–5.241)	<0.001	3.818 (2.565–5.679)	<0.001
**Whole cohort (*n* = 319)**				
Age (>60/≤60)	1.005 (0.990–1.020)	0.542	1.004 (0.988–1.020)	0.630
Gender (female/male)	0.815 (0.545–1.218)	0.318	0.788 (0.518–1.200)	0.268
Grade (G1 + G2/G3 + G4)	1.083 (0.727–1.614)	0.694	0.867 (0.568–1.323)	0.508
TNM stage (S1 + S2/S3 + S4)	2.842 (1.925–4.196)	<0.001	2.770 (1.864–4.115)	<0.001
Risk score (low/high)	2.680 (2.089–3.439)	<0.001	2.736 (2.107–3.551)	<0.001

**FIGURE 3:**
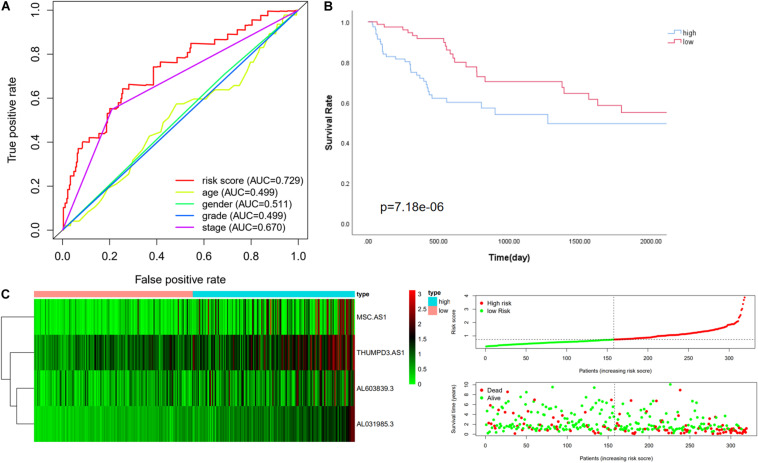
Performance evaluation of the four immune LncRNA for survival prediction in the whole cohort. **(A)** ROC curve for 5 years of OS. **(B)** Kaplan–Meier survival curve for high-risk and low-risk groups divided by the median. **(C)** Heatmaps, distribution of risk scores with survival, and statuses of patients depend on selected immune LncRNA expression.

We then further verified the performance of the four immune-related LncRNAs signature in the whole cohort and achieved similar results. The median value of the risk score in the training cohort was used to divide the whole cohort into a low-risk group (*n* = 158) and a high-risk group (*n* = 161). Consistently with this finding, Kaplan–Meier analysis showed that the OS in high-risk patients was significantly lower than that in low-risk patients (*P* value = 7.184e-06, Figure 3B). The five-year survival rates in the high-risk and low-risk groups were 41.8 and 62.3%, respectively. The specific risk score distribution, survival status of the patients, and the selected four prognostic expression profiles of LncRNAs were similar to the result for the other two cohorts (Figures 3A,C).

We compared the effectiveness of our four immune-related LncRNAs signature against two other published LncRNA signatures in HCC by using the ROC curve. The ROC analysis indicated that the risk scores calculated by their signatures were lower than ours and that the AUC values were 0.585 and 0.707 (Supplementary Figure 1). This comparison showed that our signature offers an advantage in assessing a prognosis among the clinical characteristics (Yan et al., 2019; Zhang et al., 2020).

## GSEA and PCA in the Whole Cohort

**FIGURE 4:**
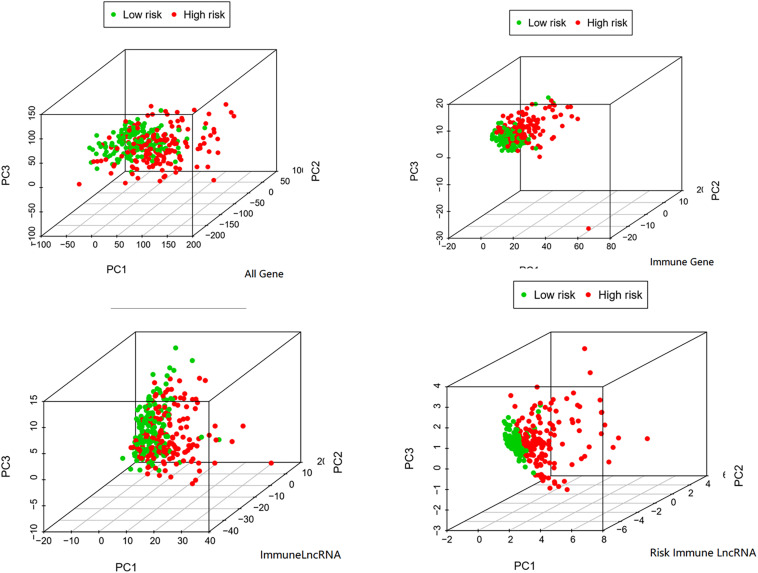
PCA among all genes, immune genes, immune LncRNA, and risk immune LncRNA.

In order to verify the significance of immune-related LncRNAs as a label to predict the prognosis of HCC patients, we performed a PCA among selected immune-related LncRNAs (Wei et al., 2019), all genes, immune-related genes, and all immune-related LncRNAs. The PCA analysis suggested that the samples screened by the four immune-related LncRNAs could clearly divide the whole group into a low-risk and high-risk group. In addition, its effect was much better than that of other signatures based on all genes, immune-related genes, or immune-related LncRNAs (Figure 4).

**FIGURE 5:**
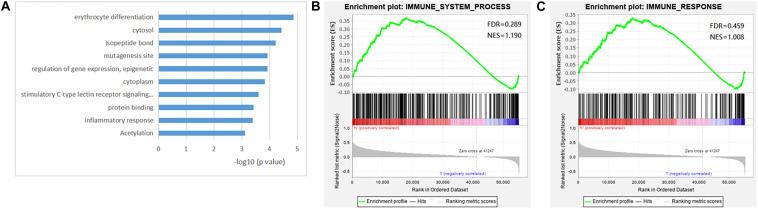
Gene set enrichment analysis (GSEA) of the four immune lncRNA signature dataset. **(A)** The top 10 biological processes and signaling pathway. GSEA validated enhanced activity of immune system progress gene set **(B)** and immune response gene set **(C)**.

Gene Set Enrichment Analysis showed a higher expression of immune LncRNA obtained from the IMMUNE_RESPONSE gene set and IMMUNE_SYSTEM_PROCESS gene set in high-risk patients, which confirmed that a high expression of relative immune-related LncRNAs may be positively associated with a poor prognosis, while the rank in the ordered gene set also implied this result (Figure 5A). GO and KEGG analysis indicated that the four immune-related LncRNAs might be connected with several biological characteristics including acetylation, inflammatory response, protein binding, stimulatory C-type lectin receptor signaling pathway, cytoplasm, regulation of gene expression, epigenetics, mutagenesis site, isopeptide bond, cytosol, and erythrocyte differentiation through the DAVID web annotation tool (Figures 5B,C; Shao et al., 2018; Yuan et al., 2018). The analysis could help us to understand the potential molecular mechanisms of the four LncRNAs in the occurrence and development of HCC (Ding et al., 2020).

## Discussion

Hepatocellular carcinoma is a well-known malignant tumor with a high morbidity and mortality. Therapy of HCC is still unsatisfactory despite the fact that great progress has been made in the technology of surgery and adjuvant therapies. Finding a significant marker that can more accurately predict the HCC prognosis will provide reliable guidance for clinical treatment. Many studies have documented that the expression of some LncRNAs is related to the prognosis of patients with various malignant tumors, including HCC. In addition, they could be used for the prediction of the prognosis of patients with better results than traditional clinical parameters (Li et al., 2017; Xie et al., 2018; Zhu et al., 2019). A single LncRNA-based prediction may lead to bias. Therefore, some researchers use comprehensive LncRNA screening to establish an evaluation equation to achieve the desired outcome (Gu et al., 2019). At the same time, studies show that immune reactions play an important role in the occurrence and development of HCC (Hou et al., 2018). We found that the immune-related LncRNAs as a signature were better at predicting the prognosis than a single LncRNA. Additionally, both PCA and GSEA proved that the immune-related LncRNA signature has a better predictive value. We compared the effectiveness of our signature with a published signature by using the ROC curve, which also supports this view (Yan et al., 2019). Our signature showed superiority in comparison with another published immune-related LncRNAs signature, considering that different screening criteria of LncRNA produce different outcomes.

The whole sample was randomly divided into a training cohort and a validation cohort. We then created the whole cohort for further validation. First, we identified 3094 immune-related LncRNAs by matching the gene ID in the IMMUNE_RESPONSE gene set and the IMMUNE_SYSTEM_PROCESS gene set obtained from the GSEA website. Single-factor Cox regression analysis screened 24 survival-related immune LncRNAs and LASSO regression analysis further identified the four most suitable immune-related LncRNAs (AL603839.3, MSC-AS1, AL031985.3, and THUMPD3-AS1) and coefficients. The risk score was used to divide patients into a low-risk group and a high-risk group. It was calculated using the coefficients and the expression of the selected immune-related LncRNAs. The AUC in the ROC of the 5-year survival rate predicted by the risk score in the validation cohort and the whole cohort was 0.727 and 0.729, respectively, which indicated that the model performed better in survival prediction than other conventional clinical parameters. Although the TNM stage also could be used as an independent factor to predict the prognosis through multivariate regression (Andreou et al., 2013), the risk score had a higher AUC and Hazard Ratio.

We then performed the GSEA to explore the role of immune-related LncRNAs in tumor behavior. The analysis showed that the four immune-related LncRNAs were significantly enriched in some biological processes and signaling pathways including acetylation, inflammatory response, protein binding, stimulatory C-type lectin receptor signaling pathway, cytoplasm, regulation of gene expression, epigenetics, mutagenesis site, isopeptide bond, cytosol, and erythrocyte differentiation (Zhou et al., 2017, 2020). It was implied that the four selected LncRNAs might be connected to some gene recombination and cell injury, thus affecting the progression and malignancy of the tumor. Studies showed that gene recombination plays an important role in eliminating the autoreactive B-cell antigen receptor, which is closely related to liver regeneration and liver failure (Behnke et al., 2018; Setz et al., 2019). Damage to the liver caused by surgery or inflammation can lead to regeneration of liver cells and may increase the risk of gene mutations, which are also closely related to the development of tumors (Yin et al., 2011; Suzuki et al., 2016). The findings are likely to be applied to the development of new targeted anti-cancer therapies if the hypothesis can be proved.

This study has several limitations. First, the equation would be more convincing if it could be verified in other databases. Actually, the LncRNAs obtained from the TCGA may not match up with those in other databases because of the use of different chips and recording methods. In other datasets that we have consulted, such as SEER, ICGC, and TANRIC, we could not find a dataset that has both the clinical data and corresponding expression of the LncRNAs. It would make the results more convincing if the effectiveness of our signature could be validated in other datasets. Second, we only performed enrichment analysis and made assumptions regarding the function of these four immune-related LncRNAs without additional research to further explore the mechanism. Third, *in vitro* experiments also could make the results more convincing. However, the sufficient survival time cannot be collected in a short period of time because the testing for expression of LncRNA requires fresh tissue samples. The further analysis of bio-information results combined with clinical data is also the direction that our follow-up research will take. Finally, although the total sample size was 319, a larger sample would produce more convincing results.

In summary, we developed a four-immune-related-LncRNA signature for the prediction of HCC prognosis after surgery. In addition, this signature might guide the use of immunotherapy in HCC.

## Data Availability Statement

Publicly available datasets were analyzed in this study, these can be found in The Cancer Genome Atlas (https://portal.gdc.cancer.gov/).

## Ethics Statement

Ethical review and approval was not required for the study on human participants in accordance with the local legislation and institutional requirements. Written informed consent for participation was not required for this study in accordance with the national legislation and the institutional requirements.

## Author Contributions

MuL and MiL: manuscript writing including the figures and tables. TW and ZC: guidance and design in figures and tables. XW and WX: manuscript writing. SS and BP: conception and design and manuscript writing. All authors: final approval of the manuscript.

## Conflict of Interest

The authors declare that the research was conducted in the absence of any commercial or financial relationships that could be construed as a potential conflict of interest. The reviewer WL declared a shared affiliation, though no other collaboration, with the authors.

## Footnotes

1 https://cancergenome.nih.gov

2 https://www.gsea-msigdb.org/gsea/msigdb/index.jsp

3 http://cran.r-project.org

## Supplementary Material

Supplementary Figure 1 The effectiveness of our signature with a published signature by using the ROC curve, A (PMID:30206981) and B (PMID:32330311).
