# Supplementary figures and images for: Four Immune-Related Long Non-coding RNAs for Prognosis Prediction in Patients With Hepatocellular Carcinoma
Source: Front Mol Biosci. 2020 Dec 8;7:566491. doi: 10.3389/fmolb.2020.566491 (PMC7752774; doi:10.3389/fmolb.2020.566491)

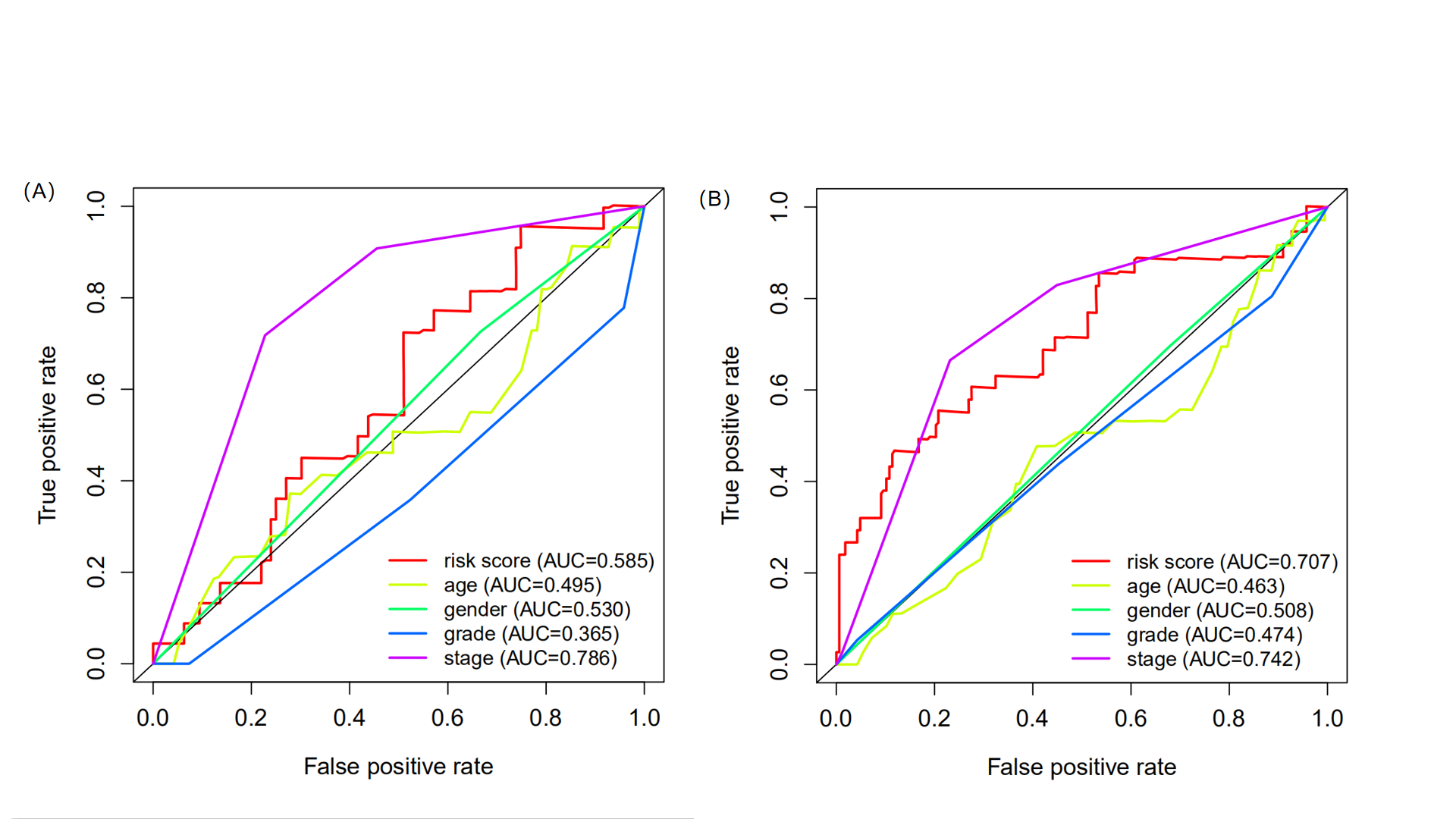

Supplement: Supplementary Figure 1 — The effectiveness of our signature with a published signature by using the ROC curve, A (PMID:30206981) and B (PMID:32330311). [file Image_1.TIF]
