# Supplementary material for: Four Immune-Related Long Non-coding RNAs for Prognosis Prediction in Patients With Hepatocellular Carcinoma
Source: Front Mol Biosci. 2020 Dec 8;7:566491. doi: 10.3389/fmolb.2020.566491 (PMC7752774; doi:10.3389/fmolb.2020.566491)
Supplement: Supplementary file 3 [file Table_1.DOCX]

Table 2. The 4 immune LncRNAs screened out by univariate Cox regression and LASSO regression analysis

| **LncRNAs** | **Hazard ratio(95%CI)** | **P value** | **coefficient** |
| --- | --- | --- | --- |
| **AL603839.3** | 2.253(1.436−3.536) | <0.001 | 0.151950286127222 |
| **MSC-AS1** | 2.088(1.414−3.083) | <0.001 | 0.101019611929165 |
| **AL031985.3** | 7.225(3.068−17.014) | <0.001 | 0.758428456611441 |
| **THUMPD3-AS1** | 2.861(1.662−4.924) | <0.001 | 0.758428456611441 |
