# Supplementary material for: Four Immune-Related Long Non-coding RNAs for Prognosis Prediction in Patients With Hepatocellular Carcinoma
Source: Front Mol Biosci. 2020 Dec 8;7:566491. doi: 10.3389/fmolb.2020.566491 (PMC7752774; doi:10.3389/fmolb.2020.566491)
Supplement: Supplementary file 4 [file Table_2.DOCX]

Table 3. Univariate and multivariate Cox regression analysis in each cohort

|  | Univariate analysis | | Multivariate analysis | |
| --- | --- | --- | --- | --- |
|  | HR (95%CI) | P value | HR (95%CI) | P value |
| Variables |  |  |  |  |
| Training cohort(n=160) |  |  |  |  |
| Age(>60/≤60) | 1.007（0.984-1030） | 0.560 | 1.000（0.976-1.025） | 0.998 |
| Gender（Female/Male） | 1.202(0.629-2.296) | 0.577 | 1.491(0.745-2.982) | 0.259 |
| Grade(G1+G2/G3+G4) | 1.015(0.558-1.847) | 0.961 | 0.717(0.374-1.374) | 0.316 |
| TNM stage(S1+S2/S3+S4) | 3.332(1.851-5.999) | <0.001 | 3.733(2.005-6.953) | <0.001 |
| Risk score(low/high) | 4.519(2.844-7.182) | <0.001 | 4.863(3.004-7.872) | <0.001 |
| Validation cohort(n=159) |  |  |  |  |
| Age(>60/≤60) | 1.007(0.984-1.030) | 0.560 | 0.997(0.974-1.021) | 0.805 |
| Gender（Female/Male） | 1.202(0.629-2.296) | 0.577 | 1.008(0.542-20185) | 0.813 |
| Grade(G1+G2/G3+G4) | 1.015(0.558-1.847) | 0.961 | 0.699(0.361-1.352) | 0.288 |
| TNM stage(S1+S2/S3+S4) | 3.332(1.851-5.999) | <0.001 | 3.365(1.808-6.263) | <0.001 |
| Risk score(low/high) | 3.627(2.510-5.241) | <0.001 | 3.818(2.565-5.679) | <0.001 |
| Whole cohort(n=319) |  |  |  |  |
| Age(>60/≤60) | 1.005(0.990-1.020) | 0.542 | 1.004(0.988-1.020) | 0.630 |
| Gender（Female/Male） | 0.815(0.545-1.218) | 0.318 | 0.788(0.518-1.200) | 0.268 |
| Grade(G1+G2/G3+G4) | 1.083(0.727-1.614) | 0.694 | 0.867(0.568-1.323) | 0.508 |
| TNM stage(S1+S2/S3+S4) | 2.842(1.925-4.196) | <0.001 | 2.770(1.864-4.115) | <0.001 |
| Risk score(low/high) | 2.680(2.089-3.439) | <0.001 | 2.736(2.107-3.551) | <0.001 |
