# Supplementary material for: Four Immune-Related Long Non-coding RNAs for Prognosis Prediction in Patients With Hepatocellular Carcinoma
Source: Front Mol Biosci. 2020 Dec 8;7:566491. doi: 10.3389/fmolb.2020.566491 (PMC7752774; doi:10.3389/fmolb.2020.566491)
Supplement: Supplementary file 5 [file Table_3.DOCX]

Table 1. Clinical factors of patients with HCC

|  | **Training cohort**  **(n=160)** | **Validation cohort**  **(n=159)** | **Whole cohort**  **(n=319)** | **P value** |
| --- | --- | --- | --- | --- |
| **Clinical factors** |  |  |  |  |
| **Gender** |  |  |  |  |
| **Male** | 49 | 51 | 100 |  |
| **Female** | 111 | 108 | 219 | 0.810 |
| **Age(year)** |  |  |  |  |
| **≤60** | 80 | 80 | 160 |  |
| **＞60** | 80 | 79 | 159 | 1.000 |
| **Grade** |  |  |  |  |
| **G1+G2** | 98 | 100 | 198 |  |
| **G3+G4** | 62 | 59 | 121 | 0.818 |
| **TNM stage** |  |  |  |  |
| **I+II** | 120 | 116 | 236 |  |
| **III+IV** | 40 | 43 | 83 | 0.703 |
| **Survival status** |  |  |  |  |
| **Alive** | 114 | 101 | 215 |  |
| **Dead** | 46 | 58 | 104 | 0.153 |
